# Effect of the ORMOSIL Used for the Functionalization of MSNs in the Removal of Anionic Contaminants from Sugarcane Processing Wastewater

**DOI:** 10.3390/nano16060368

**Published:** 2026-03-17

**Authors:** William A. Talavera-Pech, Carlos A. Chan-Keb, Ángel A. Bacelis-Jiménez, Judith Ruiz-Hernández, Valentina Aguilar-Melo, Claudia M. Agraz-Hernández

**Affiliations:** 1 Instituto de Investigación en Corrosión y Preservación de Materiales, Universidad Autónoma de Campeche, Av. Héroe de Nacozari 480, San Francisco de Campeche 24079, CAM, Mexico; watalave@uacam.mx (W.A.T.-P.); vaguilar@uacam.mx (V.A.-M.); 2 Facultad de Ciencias Químico-Biológicas, Universidad Autónoma de Campeche, Avenida Ing. Humberto Lanz Cárdenas S/N, Colonia Ex Hacienda Kalá, San Francisco de Campeche 24085, CAM, Mexico; judiruiz@uacam.mx; 3 Facultad de Matemáticas, Universidad Autónoma de Yucatán, Anillo Periférico Norte, Tablaje Catastral 13615, Colonia Chuburná Hidalgo Inn, Mérida 97119, YUC, Mexico; angel.bacelis@correo.uady.mx; 4 Instituto EPOMEX, Universidad Autónoma de Campeche, Av. Héroe de Nacozari #480, Campus 6 de Investigaciones, San Francisco de Campeche 24029, CAM, Mexico

**Keywords:** mesoporous silica nanoparticles, organically modified silanes, anion removal, wastewater

## Abstract

Water pollution from the sugar industry is a significant environmental problem as it generates effluents containing organic compounds, solids, nutrients, and chemicals such as H_3_PO_4_, SO_2_, and Ca (OH)_2_. Mesoporous silica nanoparticles (MSNs) are a promising option for its treatment, due to their high surface area, and ease of functionalization using organically modified silanes (ORMOSIL) improving its adsorption of contaminants. The objective of this study is to remove anions (Cl^−^, SO_4_^2−^, NO_2_^−^, NO_3_^−^) from the wastewater of a sugar mill in Campeche, Mexico and improve its physicochemical parameters (conductivity, turbidity, dissolved oxygen) using MSNs functionalized with 3-aminopropyltriethoxysilane (MSNs-APTES) or 3-(2-aminoethylamino)propyltrimethoxysilane (MSNs-3-2-A). The synthesized materials were characterized by FTIR and XPS analyses, which confirmed the incorporation of amino functional group and that MSNs-APTES exhibited a stronger N1s signal, indicating greater surface accessibility of amino groups. However, a partial surface masking under complex aqueous conditions was revealed. In contrast, MSNs-3-2-A showed lower apparent surface exposure of amino groups maintaining a more stable functional presence after exposure, likely due to its diamine structure promoting more confined interactions within the mesoporous framework. The results of removing anions and physicochemical parameters of wastewater exposed to MSNs indicate that treatments with MSNs-APTES and MSNs-3-2-A were able to significantly reduce the concentrations of SO_4_^2−^, NO_2_^−^ and NO_3_^−^ anions, but not able to reduce the chloride ion. A decrease in turbidity and an increase in dissolved oxygen were also observed. Then, both materials proved to be functional and stable in contact with wastewater, demonstrating their potential for environmental remediation, particularly for the removal of anionic contaminants from sugar industry effluents.

## 1. Introduction

Water pollution from sugar industry effluents represents an environmental problem that has been a cause for concern in various regions of the world [1]. Sugar production involves the processing of sugarcane, which requires the use of large volumes of water, and during this process, several by-products and liquid effluents are generated, which can be harmful to the environment if there is no existing correct final disposal method [1,2]. Wastewater from sugar mills typically contains organic compounds, suspended solids, nutrients such as nitrogen and phosphorus, and sometimes chemicals used during sugarcane processing, such as H_3_PO_4_, SO_2_, and Ca(OH)_2_ [1,3]. Sugar mill wastewater discharges lead to decreased water quality, eutrophication of water bodies (increased nutrients that lead to overgrowth of algae and aquatic plants), and detrimental impacts on aquatic ecosystems [3,4].

Because of this, sugar mills often implement wastewater treatment systems to reduce the pollutant load before releasing the effluent into the environment. Common treatment methods include biological, physical, and chemical processes. Similarly, each country’s environmental regulations and local regulations play an important role in the management of water pollution by sugar mills, which establish standards and limits for industrial discharges [2,4].

A recently evaluated wastewater treatment option focuses on mesoporous silica nanoparticles (MSNs) [5]. MSNs are silica nanoparticles obtained by the sol–gel synthesis method, using surfactants, such as templates and silicon precursors in alkaline media. Through this synthetic methodology it is possible to obtain, depending on the synthesis conditions, particles of approximately 100 nm in diameter and with different mesopores order structures. The main reasons for the use of MSNs in wastewater remediation are, on the one hand, that they have a high specific surface area, close to 1000 m^2^ g^−1^, which is related to a large number of points where contaminants can be adsorbed, and on the other hand, the ease with which they can be functionalized, that is, with which its surface can be doped with functional groups, using organically modified silanes (ORMOSIL) during its synthesis or in a post-synthetic procedure, improving their affinity for contaminants of various types [6]. For example, the MSNs materials were functionalized by co-condensation or post-synthesis procedure for the removal of Reactive Black 5, which is an anionic azo-dye (N=N–) [7], and for the removal of metals in wastewaters such as Cr (IV) ion [8].

Thus, functionalized MSNs have been evaluated for the removal of different types of contaminants. For example, they have been doped with many chemical groups for the removal of metal ions such as Ag^2+^ [9], Fe^3+^ [10], Ni^2+^ [11], Co^2+^ [12], among many others [13]. On the other hand, they have also been functionalized with histidine for the removal of cationic dyes, such as methylene blue, and anionic dyes, such as phenol red, achieving adsorption capacities of 60 mg g^−1^ for methylene blue, and 50 mg g^−1^ for phenol red at the maximum equilibrium [14]. Another type of functionalization explored was the incorporation of amino groups, using 3-aminopropyltriethoxysilane as the ORMOSIL, for the removal of the azo-type dye black reagent 5, obtaining a maximum adsorption capacity of 436 mg g^−1^ [7].

The sugar industry, being a key pillar in the Mexican economy, plays an essential role in the production of sugar and its derivatives, contributing significantly to the country’s economic development [3]. However, this sector is not exempt from environmental challenges, and concern about pollution from its industrial processes has generated attention in recent years, associated with the final disposal of effluent and waste generated by sugar factories [2].

Therefore, the objective of this study was to remediate sugarcane processing wastewater by removing anions such as SO_4_^2−^, NO_2_^−^, and NO_3_^−^ by applying MSNs functionalized with two different amino-bearing ORMOSILs. Then, the evaluation of how different functionalization groups influence anion removal efficiency and the subsequent improvement of water physicochemical parameters such as conductivity, turbidity, and dissolved oxygen level.

## 2. Materials and Methods

### 2.1. Collection of Wastewater Samples from Sugarcane Processing

**Figure 1:**
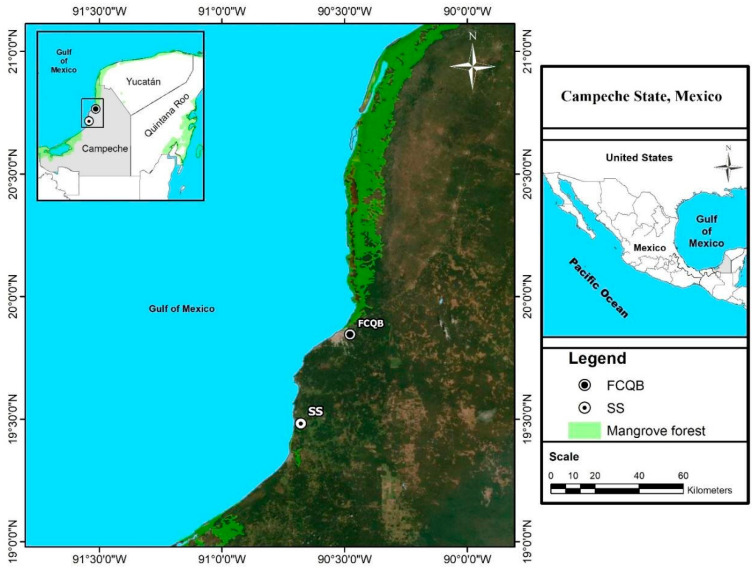
Geographical location of the wastewater collection and site of development of the experiment (FCQB).

The collection of wastewater samples was carried out in August 2024 near the sugar mill located at coordinates 19°28′ 57.041″ N and 90°40′ 32.465″ W (Figure 1), in the state of Campeche, Mexico, which is the place where wastewater is discharged. The samples were collected into 3500 mL bottles. Later, they were transferred to the Laboratory of Physicochemical Analysis of Water at the Faculty of Chemical–Biological Sciences of the Autonomous University of Campeche. The state of Campeche is in Southern Mexico, with 523 km of coastline along the Gulf of Mexico. Campeche has a tropical savanna climate, classified as Aw by the Köppen–Geiger system, with heavy rain falling during the summer from June to October. Precipitation increases from the northwest (600 mm/year) to the southeast (1400 mm/year) [15]. Sugar production at the sugar mill occurs during the annual harvest season, which includes harvesting and milling sugarcane. This harvest season typically takes place between November and August of each year, coinciding with the availability of cane ready for processing and favorable weather conditions for the sugar industry.

### 2.2. Determination of the Physicochemical Parameters of Wastewater

The physiognomic parameters of wastewater from the processing that were measured were: turbidity, dissolved oxygen, pH, conductivity, Cl^−^, NO_2_^−^, NO_3_^−^, and SO_4_^2−^. Water turbidity was determined using a portable turbidimeter model HI93703 (Hanna Instruments^®^, Woonsocket, RI, USA). Dissolved oxygen (DO) in the water was measured with a DO9100 electrode (Apera Instruments, Columbus, OH, USA), and pH was measured with a HACH HQ40d portable multimeter (Loveland, CO, USA). The samples collected at the sampling site were stored at 4 °C until processing. All samples were processed within 1 week. Processing consisted of allowing the water samples to reach room temperature and filtering them (using 47 mm GF/A glass microfiber filters) before its spectrophotometry analysis. Each chemical analysis was performed in triplicate. Subsequently, the absorbance of each sample was measured using a 6500 UV-Vis spectrophotometer (Jenway, Stafford, UK). Inorganic anions were determined using standardized methods for water analysis. Chloride (Cl^−^) was quantified by argentometric titration (Mohr method) using standardized silver nitrate and potassium chromate as the indicator, with the endpoint determined by the formation of silver chromate. The concentration of nitrites (NO_2_^−^) was determined by UV-Vis spectrophotometry using the Griess reaction, with readings at 540–545 nm and quantification via an external calibration curve. Nitrate (NO_3_^−^) was analyzed by direct UV spectrophotometry at 220 nm with correction for organic interference at 275 nm. Sulfate (SO_4_^2−^) was determined by the turbidimetric method based on precipitation as barium sulfate (BaSO_4_) after the addition of barium chloride, and turbidity was measured at approximately 420 nm. All analyses were performed according to standardized methods (APHA Standard Methods), including external calibration curves, analytical blanks, and replicates to ensure measurement precision and accuracy [16].

### 2.3. Synthesis and Functionalization of Nanoparticles

The synthesis of nanoparticles was carried out by the sol–gel method using tetraethyl orthosilicate (TEOS) as the silicon precursor and cetyltrimethylammonium bromide (CTAB) as the cationic surfactant [17,18]. For this, 0.2 g of CTAB was weighed and added to a balloon flask containing 96 mL of deionized water and 0.7 mL of 2 M NaOH. This mixture was heated to 80 °C, and at that temperature, 1.4 mL of TEOS was added and heated for 2 h. Subsequently, the CTAB was extracted from the pores by suspending 200 mg of the nanoparticles obtained in 50 mL of a 40 mM ammonium chloride solution. This mixture was sonicated at 60 °C for 2 h. Finally, the mesoporous silica nanoparticles were functionalized separately using two organically modified silanes in accordance with previous procedures [19,20]: (1) 3-aminopropyltriethoxysilane (samples called MSNs-APTES) and (2) 3-2 aminoethyl aminopropyl triethoxysilane (samples called MSNs-3-2-A); for this, the MSNs were resuspended in toluene, followed by the addition of 15 mol% of the respective ORMOSIL precursor (respective to TEOS used for the synthesis). The resulting solution was heated to reflux for 6 h, then vacuum filtered.

### 2.4. Characterization of Nanoparticles

To corroborate the correct synthesis of MSNs of MCM-41-type, the obtained materials were characterized trough Scanning Electron Microscopy (SEM), and Small Angle X-Ray scattering (SAXS). For this, SEM was conducted on a JEOL JSM-7601F microscope (JEOL Ltd., Tokyo, Japan) with 10 kV of acceleration voltage. The powders were placed inside glass Mark-tubes for the SAXS measurement. The SAXS patterns were recorded for 300 s with a S3-MICRO SAXS camera system (HECUS X-ray Systems, Graz, Austria). Cu Ka radiation of wavelength 1.542 A ˚ was provided by a GeniX microsource X-ray generator (XENOCS, Grenoble, France) operating at 25 kV and 0.25 mA. To corroborate the functionalization of the MSNs with each of the ORMOSILs, the bare and functionalized MSNs were analyzed by Fourier transform infrared spectroscopy. For this, a Thermo Scientific™ Nicolet™ iS™5 FT-IR instrument (Thermo Fisher Scientific, Waltham, MA, USA) was used with the ATR (Attenuated Total Reflection) technique over the spectral range of between 4000 and 400 cm^−1^, with 100 scans at a resolution of 4 cm^−1^. To determine the elemental composition of the surface of the MSNs, an X-ray photoelectron spectroscopy analysis was carried out, using a Thermo Scientific K-Alpha equipment (Thermo Fisher Scientific, Waltham, MA, USA) equipped with a monochromatic Al K-Alpha source (1486.6 eV) with an energy of 100 eV and a step of 1 eV. XPS samples were taken before and after exposure to wastewater.

### 2.5. Removal of Anions with Functionalized Nanoparticles

For the analysis of anion removal, 50 mg of MSNs-APTES or MSNs-3-2-A were suspended in 50 mL of wastewater. This mixture was sonicated for 2 h at room temperature, then left under mechanical agitation for 24 h. At the end of the 24 h period, the samples were centrifuged at 9500 RPM for 30 min, and the supernatants were collected for further anion-content analysis, while the nanoparticles were stored for post-exposure XPS analyses described in the previous section.

### 2.6. Anion Removal Efficiency

The removal efficiency of nitrites, nitrates, and sulfates was calculated as a step for the evaluation of the efficiency of the anion removal, following the formula:

% R = (C E − C S)/C E × 100

where % R = removal efficiency of the anions, E C = concentration of effluent inlet, and C S = effluent outlet concentration.

## 3. Results and Discussion

### 3.1. Physicochemical Characterization of Pristine Nanoparticles

**Figure 2:**
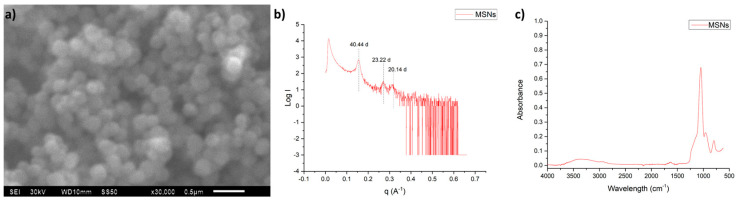
Physicochemical characterization of pristine MSNs through (**a**) SEM, (**b**) SAXS, and (**c**) FTIR analysis.

In the SEM image of MSNs, Figure 2a, it is possible to observe a spherical shape for the obtained nanoparticles, with a diameter of about 100 nm, which is characteristic for MCM-41-type silica materials.

Small angle X-Ray scattering (SAXS) analysis was conducted in order to corroborate the MCM-41 honey characteristic ordering of pores. The results presented in Figure 2b indicate the obtention of three main peaks associated with MCM-41-type materials, i.e., the [d100] peak at 40.44 angstroms, the peak corresponding to [d110] at 23.22 Angstroms and the peak for [d200] at 20.14 angstroms. The appearance of these peaks in those specific positions clearly confirms the obtention of MCM-41-type MSNs.

The FTIR spectra of as-obtained MSNs, presented in Figure 2c, reveal only the presence of peaks associated with a SiO_2_ network, which are a broad band at 3300 cm^−1^ corresponding to Si-OH groups, and an intense peak close to 1080 cm^−1^ which corresponds to Si–O–Si bonds. Also, the lack of peaks at 2850 and 2930 cm^−1^ of methyl and methylene groups confirms the correct elimination of the surfactant used for the synthesis of MCM-41-type MSNs.

### 3.2. Physicochemical Characterization of Functionalized Nanoparticles

**Figure 3:**
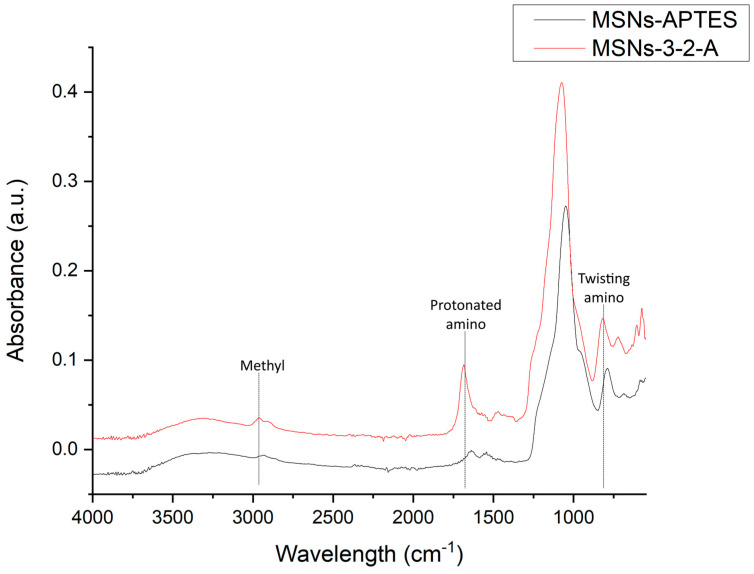
FTIR spectra of samples functionalized with 3-aminopropyltriethoxysilane (APTES) and 3-(2-aminoethylamino)propyltrimethoxysilane.

Figure 3 shows the FTIR spectrum of mesoporous silica nanoparticles modified with APTES and 3-2 aminoethyl aminopropyl triethoxysilane. In the spectrum, the characteristic peaks of the SiO_2_ lattice can be observed for both samples, i.e., the characteristic peak of the asymmetrical stretch vibration of the Si–O–Si bonds positioned at 1081 cm^−1^; also, a shoulder-shaped peak of the main peak located at 946 cm^−1^ corresponding to the bending vibration of the Si–OH bond is observed [21]. In addition to these peaks that are common for silica nanoparticles, functionalization with the two different ORMOSILs with amino groups feature typical peaks of functionalized structures, such as 2930 cm^−1^ which is a characteristic peak for methyl, and a peak at approximately 800 cm^−1^ associated with the twisting vibration of amino groups, finally a peak close to 1660 cm^−1^, which is a peak associated with protonated amino groups [22]. These results corroborate the correct functionalization of MSNs with amino groups; however, the last three peaks mentioned are found with a greater intensity in the MSNs-3-2-A sample, which corresponds to the greater presence of both amino and methylene groups in this sample, since it has two nitrogen atoms and an ethyl chain and a propyl chain in its structure, compared to APTES which has only one nitrogen atom and one propyl chain in its structure.

X-ray photoelectron spectroscopy (XPS) was employed to evaluate the surface elemental composition and chemical state of mesoporous silica nanoparticles (MSNs) functionalized with APTES and 3-(2-aminoethylamino)propyltrimethoxysilane (3-2-A). These organosilanes were selected to introduce amine functionalities, enhancing the affinity of the materials toward anionic contaminants such as nitrates and sulfates through electrostatic interactions [23,24].

**Figure 4:**
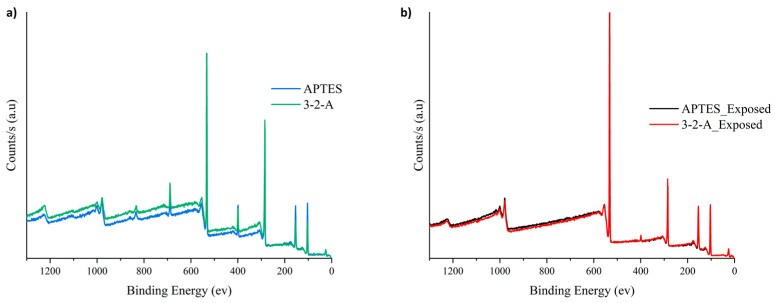
Survey spectra of both materials: (**a**) before and (**b**) after exposure to wastewater.

**Figure 5:**
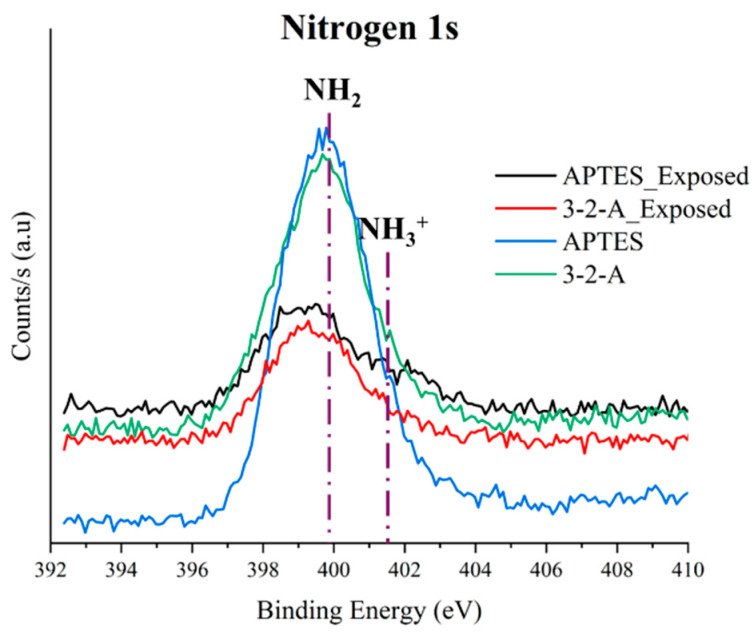
High-resolution XPS spectra for N1s before and after exposure for (APTES) and 3-(2-aminoethylamino)propyltrimethoxysilane.

**Figure 6:**
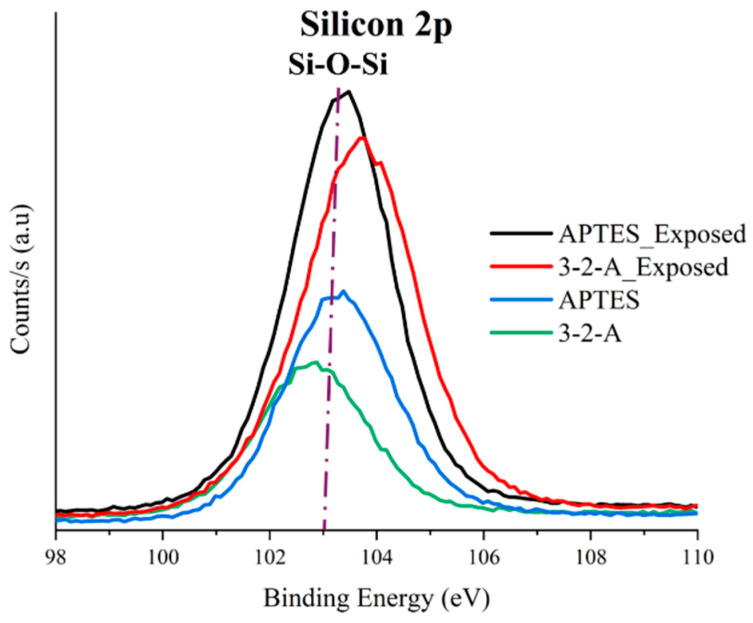
High-resolution XPS spectra for Si2p before and after exposure for (APTES) and 3-(2-aminoethylamino)propyltrimethoxysilane.

**Figure 7:**
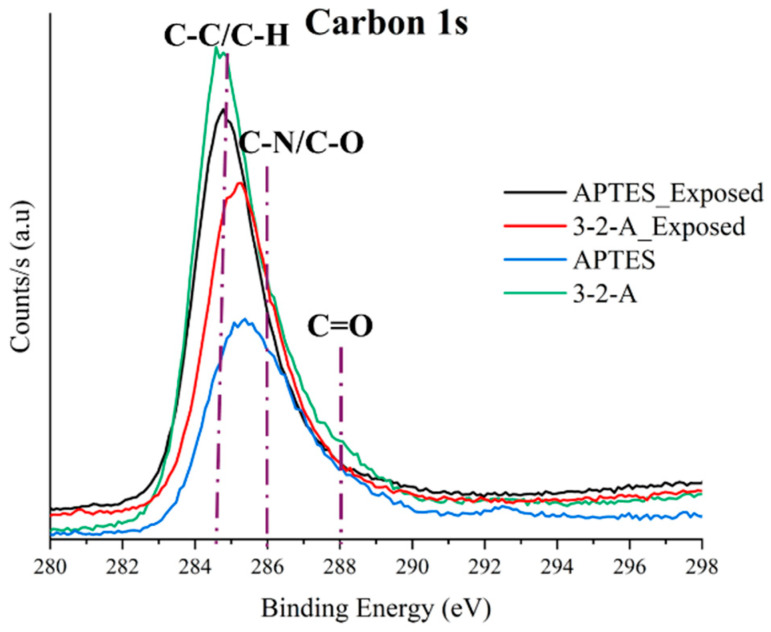
High-resolution XPS spectra for C1s before and after exposure for (APTES) and 3-(2-aminoethylamino)propyltrimethoxysilane.

Survey (Figure 4) and high-resolution spectra (Figure 5, Figure 6 and Figure 7) were analyzed for both materials before and after exposure to sugarcane processing wastewater. Since the analysis was conducted on powder samples, the results reflect the outermost surface (~10 nm), where functional group accessibility, structural rearrangements, and the adsorption of organic matter from the effluent play a critical role in determining the detected composition.

In the case of MSNs functionalized with APTES, the initial N1s signal (~399 eV) confirmed the successful incorporation of surface amine groups, with a relatively high nitrogen concentration (8.86%) and a low Si/N ratio (2.50), indicating a high density of accessible active sites. However, after wastewater exposure, a pronounced decrease in nitrogen content to 2.24% was observed, accompanied by a substantial increase in the Si/N ratio to 10.07 (Table 1). This behavior is consistent with the formation of an adsorbed organic layer that masks the underlying functional groups, rather than complete leaching of the silane. This interpretation aligns with previous reports on hybrid adsorbents exposed to complex industrial matrices [25].

Additionally, a shift in the N1s binding energy toward higher values (~401.5 eV) was observed after treatment, indicating protonation of the amine groups (-NH_3_^+^). This confirms that electrostatic interactions between positively charged surface sites and anionic species in the wastewater govern the adsorption mechanism under these conditions [3].

In contrast, MSNs functionalized with 3-2-A exhibited a lower initial nitrogen content (5.90%), which can be attributed to differences in molecular structure and spatial arrangement within the mesoporous framework. Despite this lower initial loading, the reduction in N1s intensity after exposure was less pronounced compared to APTES, indicating a more stable functional layer. The diamine structure of 3-2-A provides multiple nitrogen centers, which enhances resilience against surface masking and preserves functional activity under competitive adsorption conditions [26].

This behavior is consistent with FTIR results, where stronger signals associated with C–H and N–H vibrations suggested a higher organic content and structural stability. Furthermore, the partial confinement of 3-2-A groups within mesoporous channels may contribute to protecting active sites from complete surface coverage by organic macromolecules, allowing continued interaction with target contaminants [25].

The absence of a detectable Cl2p signal (~198 eV) after treatment indicates that chloride adsorption is not a dominant mechanism for either material. However, the significant increase in the C/O ratio (from 0.83 to 2.42 in the case of 3-2-A) confirms the adsorption of carbon-rich organic species from wastewater, supporting the surface masking hypothesis. The presence of F1s in the initial samples (2.81–3.24%) and its disappearance after treatment is attributed to residual contamination from PTFE-based materials used during synthesis, which are removed upon washing [27].

Comparative analysis of both functionalization strategies reveals distinct surface interaction pathways. While APTES provides a high initial density of accessible amine groups, it is more susceptible to surface fouling under complex wastewater conditions. In contrast, the 3-2-A functionalization, characterized by its diamine structure, promotes a more stable and resilient adsorption environment, maintaining functional performance despite partial surface masking [28].

Overall, the combined XPS and FTIR results demonstrate that the molecular architecture of the silane coupling agent plays a decisive role in determining not only the initial functionalization efficiency but also the long-term accessibility and stability of active sites. These findings highlight the potential of diamine-functionalized MSNs as robust materials for the treatment of complex industrial effluents, such as those generated in the sugar industry [26].

### 3.3. Concentration and Values of Physicochemical Parameters of Wastewater

**Figure 8:**
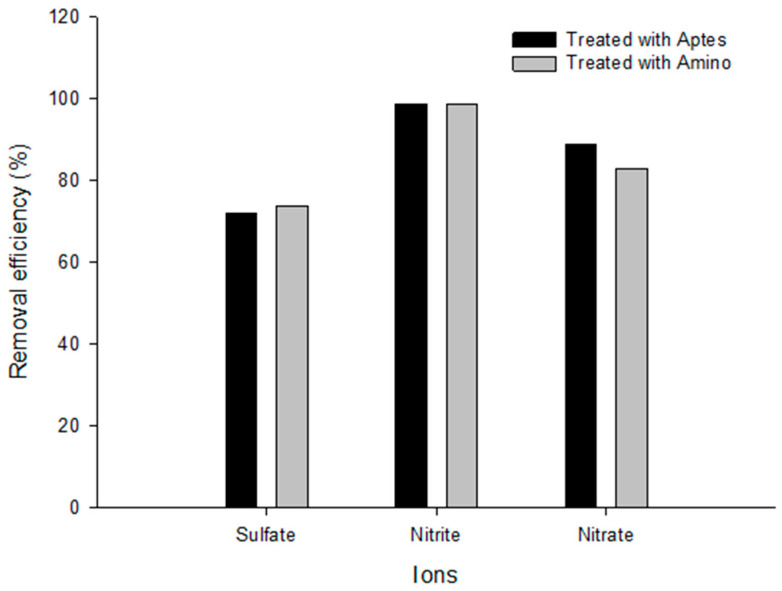
Removal efficiency of sulfates, nitrites, and nitrates using two types of treatments: Aptes and Amino from sugarcane processing wastewater.

The results show that both functionalized MSNs (APTES and 3-2-A) used as wastewater treatments have similar efficiencies in removing sulfates, nitrites, and nitrates from sugarcane processing wastewater. The MSNs-APTES treatment achieved removal efficiency of 72.1% for sulfates, 98.72% for nitrites, and 88.67% for nitrates; while the MSNs-3-2-A treatment showed values of 73.76%, 98.73%, and 82.82%, respectively (Figure 8). When comparing both methods, a marginal increase, close to 1%, in the removal of sulfates and nitrites by MSNs-3-2-A treatment is observed, which suggests a slight improvement attributable to a greater availability of protonable functional groups, capable of establishing more favorable electrostatic interactions with these anions. However, in the case of nitrate, the MSNs-APTES treatment exhibited approximately 6% higher efficiency, which could be due to differences in the orientation, accessibility, or effective density of organosilane groups that increase the affinity for NO_3_^−^ (Table 2).

These results are consistent with those reported by [24], who observed high nitrite and nitrate removal efficiencies in real systems when using materials with functional groups that enhance the uptake of highly mobile anions. Similarly, [29] demonstrated that functionalization with APTES can markedly increase oxyanion adsorption due to the three-dimensional conformation of 3-aminopropyltriethoxysilane, which modulates electrostatic interaction and surface access to active sites. Likewise, the moderate efficiency observed for sulfates in this study is consistent with previous reports that indicate the complexity of removing SO_4_^2−^ in real industrial matrices due to their high hydration radius and competition with other anions present [30].

Overall, the findings suggest that both treatments perform competitively with the literature and that the choice of the most appropriate method may depend on the target anion and the composition of the residual matrix. The observed increase in sulfates and nitrites by MSNs-3-2-A treatment may be advantageous in applications where these anions are a priority, while APTES treatment may be more appropriate in situations where nitrate predominates. These results provide relevant evidence for the design of functionalized materials aimed at advanced agro-industrial wastewater treatment.

## 4. Conclusions

MSNs were synthesized and functionalized with two different ORMOSIL reactants in order to add amino groups to their surfaces using two different ORMOSIL precursors, i.e., APTES and 3-2-A, and then were used to remove anions and to change the physicochemical parameters of wastewater from the sugar industry. The synthesized silica nanoparticles are of the MCM-41-type, as demonstrated by SEM, SAXS and FTIR that they possess a morphology, hexagonal pore structure and chemical composition corresponding to this type of nanoparticles. For the functionalized materials, the FTIR results corroborate the presence of amino groups in both samples MSNs-APTES and MSNs-3-2-A, as both samples present the peak at 1660 cm^−1^ corresponding to protonated amino groups, which can have good interaction with the anions in the wastewater.

The X-ray photoelectron spectroscopy (XPS) results demonstrate that mesoporous silica nanoparticles (MSNs) functionalized with different amino ORMOSILs exhibit distinct surface chemistries, leading to differences in amine group accessibility and interaction behavior with anionic species present in industrial wastewater. MSNs-APTES nanoparticles showed a higher N1s signal intensity, indicating greater surface availability of amino groups, which favors rapid electrostatic interactions with dissolved anions. However, a significant decrease in nitrogen content after treatment suggests partial surface masking under complex wastewater conditions. In contrast, MSNs-3-2-A exhibited lower apparent surface exposure of amino groups but maintained a more stable functional presence after treatment. This behavior is attributed to the diamine structure, which may promote more confined or selective interactions within the mesoporous framework.

Overall, both materials remain functional in wastewater environments but operate through different interaction pathways; MSNs-APTES favors higher initial surface activity, whereas MSNs-3-2-A provides enhanced stability against surface fouling. These findings highlight the importance of molecular design in supporting the performance of amino-functionalized MSNs and their potential as versatile materials for the removal of anionic contaminants in complex industrial effluents.

The MSNs-APTES and MSNs-3-2-A treatments demonstrated high efficiency in removing sulfates, nitrites, and nitrates from sugarcane processing wastewater, achieving approximately 99% nitrite removal. Although both methods have comparable performance, a slight advantage was observed in the MSNs-3-2-A treatment for sulfates and nitrites, and in MSNs-APTES for nitrates, indicating that the arrangement of the functional groups influences the adsorption of anions. These results confirm that MSNs functionalized with amino groups are promising materials for wastewater treatment, although it is recommended to evaluate their efficacy under more complex conditions and their regenerative capacity.

## Figures and Tables

**Table 1:** Atomic concentrations (%) from high resolution XPS and calculated Si/N and C/O ratios.

	Element (%)	O1s	Si2p	C1s	N1s	F1s	Cl2p	Si/N	C/O
MSNs + APTES	Before	29.18	22.14	37.01	8.86	2.81	0.00	2.50	1.268
	After	40.75	22.56	34.45	2.24	0.00	0.00	10.07	0.845
MSNs + 3-2-A	Before	23.23	11.07	56.26	5.9	3.24	0.30	1.88	2.422
	After	38.65	26.93	32.24	1.99	0.20	0.00	13.55	0.834

**Table 2:** Concentration of the physicochemical parameters of the wastewater from sugarcane processing before and after treatment with MSNs-APTES and MSNs-3-2-A.

Physicochemical Parameters	Untreated Wastewater	Treated Wastewater (MSNs-APTES)	Treated Wastewater (MSNs-3-2-A)
Turbidity (UTN)	292.5 ± 116	52 ± 0.14	145 ± 5.65
Dissolved oxygen (mg/L)	3.5 ± 0.16	7495 ± 0.28	7.43 ± 0.02
pH	3.8 ± 1.68	4 ± 0.07	2.43 ± 0.056
Conductivity (μs)	1621 ± 17	1480 ± 1	1638.5 ± 14
Cl^−^ (mg/L)	559 ± 57	866.68 ± 57	843.99 ± 25
SO_4_^2−^ (mg/L)	552 ± 113	154.08 ±17	144,835 ± 5.35
NO_2_^−^ (mg/L)	5.87 ± 0.25	0.075 ± 0.01	0.074 ± 0.002
NO_3_^−^ (mg/L)	4.78 ± 0.27	0.542 ± 0.02	0.821 ± 0.014

## Data Availability

Data are contained within the article.
